# Remarks on the Monesia

**Published:** 1840-04-04

**Authors:** Joseph G. Nancrede

**Affiliations:** Walnut street


*Remarks on the Monesia.* By Joseph G. Nancrede, M. D.

To the Editors of the Medical Examiner.

*Gentlemen*:—With the intention of extending a knowledge of the properties of monesia, and of thus rendering practically useful, to our community, the very interesting paper of Dr. Martin Saint Ange, a translation of which appeared in the last number of your journal, I beg leave, through the medium of its pages, to acquaint the medical profession, that a portion of monesia, recently received from Paris, has been placed in the hands of Mr. F. Brown, corner of Fifth and Chesnut streets, where it can be obtained by those who may wish to prescribe it.

The preparations which have reached me, and which are to be obtained here, are, 1, the aqueous extract; 2, a syrup containing about six grains of the extract to the ounce; 3, a hydro-alcoholic tincture, containing about thirty-two grains to the ounce; 4, an ointment, containing one-eighth of its weight of extract.

While on this subject, I may be permitted to state, that having had in my possession for about a week, this new substance accompanied by the *Gazette Médicale de Paris*, of 19th October, 1839, in which I read Dr. Martin Saint Ange's original paper, and also the *Notices sur le Monesia* by M. Bernard Derosne, to whom is due the credit of having introduced the new article to the notice of the profession, and who, in conjunction with M. O'Henry, has made it a chemical analysis, I naturally felt desirous of being the first on this side of the Atlantic to test the properties of so interesting a remedy.

Accordingly I have exhibited it in five cases; and though it may be premature, in so short a period, to form any positive conclusions, yet I may be allowed to state, that, during the short period of its use in my hands, the medicine has realised the expectations held out by its friends in Paris.

I will merely add for the present, that I have exhibited the extract internally in the form of pills, in three cases, of which the first was diarrhœa, of long standing. Here its tonic and astringent qualities evidently arrested the disease. The two next cases were those of patients labouring under dysmenorrhœa; in both which, the symptoms have been materially improved under its exhibition. The fourth case is one of ulceration of the mouth, involving the tongue, of some week's duration, which I have treated by a solution of one part of the tincture, to five of water, as a local application, for the three last days, I think, with some benefit to my patient. The fifth case, is one of scrofulous ulceration, upon which I have applied the powdered extract twice. As yet, I cannot discover any change for the better.

These cases of the administration of the monesia, in several of its preparations, are recent, the oldest being only of a week's date, yet the results are so far encouraging, as to induce the wish that the American Medical Profession, which has now an opportunity of becoming acquainted with monesia, may fairly and fully test the properties of this new addition to the Materia Medica.

*Walnut street, April*, 1840.

